# Biocheese: A Food Probiotic Carrier

**DOI:** 10.1155/2015/723056

**Published:** 2015-02-23

**Authors:** J. M. Castro, M. E. Tornadijo, J. M. Fresno, H. Sandoval

**Affiliations:** ^1^Department of Molecular Biology, University of León, Campus de Vegazana, 24071 León, Spain; ^2^Department of Food Science and Technology, University of León, Campus de Vegazana, 24071 León, Spain

## Abstract

This review describes some aspects related to the technological barriers encountered in the development and stability of probiotic cheeses. Aspects concerning the viability of probiotic cultures in this matrix are discussed and the potential of cheese as a biofunctional food carrier is analyzed, outlying some points related to health and safety. In general, the manufacture of probiotic cheese should have little change when compared with the elaboration of cheese in the traditional way. The physicochemical and technological parameters influencing the quality of these products have also to be measured so as to obtain a process optimization.

## 1. Introduction

Functional foods are those which contain some health-promoting components which go beyond the traditional nutrients [1]. One way in which foods can be modified to become functional is by adding probiotics. A probiotic food is a processed product which contains viable probiotic microorganisms in a suitable matrix and in sufficient concentration [2].

Traditionally, the most popular food delivery systems for probiotic cultures have been freshly fermented dairy foods such as yoghurts and fermented milks. However, their survival and viability may be adversely affected by processing conditions as well as by the product environment and storage conditions. From a regulatory point of view, probiotic population must be stated on the product label [1]. Most current national legislations establish minimum viable quantities of 10^6^-10^7^ CFU/g or CFU/mL of probiotic cultures present in the food taking into account a daily consumption of 100 g or 100 mL. In order for a cheese to be recognized as probiotic, appropriate probiotic added microorganisms have to retain quantity and quality throughout the process steps involved in the manufacture, which is not all that easy, beginning with the fact that competition will more than likely be exerted by starter cultures and ending with the challenge to obtain a proper delivery in the gastrointestinal tract (GIT).

For products such a cheese where the probiotic displays an active metabolism the stability depends on the inherent aptitudes of the strain involved and on the physical properties of the matrix. The matrix has a large impact on the probiotic viability and shelf-life.

Many compositional and process factors significantly affect the viability of probiotics in cheese including the kind and the amount of probiotic inoculation, flavouring supplementation, microbiota competition, possible presence of bacteriocines or other antimicrobials, pH, redox potential, incubation and storage temperature, salt and water activity and packaging materials, and other factors.

It is apparently clear from the data collected that survival fitness is linked to a particular strain and not to a particular species or genus. Therefore, it is crucial to study case by case to see whether the characteristics are properly maintained in the cheese matrix or not. An illustrative generic example could be* Lactococcus lactis* ssp.* lactis*, which is a candidate probiotic for use in aquaculture [3]. The significative higher halotolerance in strains from marine fish compared to those used as cheese starter indicates that each strain has adapted to its particular environment confirming the need to carry out a careful strain selection depending on the purpose. It is hard to visualize how the behaviour of a culture will turn out. Therefore, in-depth studies have to be carried out to ensure proper characteristics. Hence, it is mandatory to confirm the stability in order to ensure that the characteristics are retained.

The incorporation of probiotics into a wide range of food products is conditioned by the food matrix. Probiotic bacteria have been incorporated into a wide range of foods, including dairy products (such as yoghurt, cheese, ice cream, desserts, cultured milks, or pasteurized unfermented milk). Although dairy products are currently the most common platform for delivery of probiotics we should remember that probiotics are also sold in nondairy products (such as chocolate, cereals, and juices) and other different non-food-related formulations. Either way the viability of cells is of crucial importance because they have to stay alive until reaching their action site. Many reports indicated that there is poor survival of probiotic bacteria in products containing free probiotic cells [5].

There are still people who debate whether the beneficial probiotic cultures actually survive in cheese. Many cheeses are presently being developed as probiotic and scientific data show that the probiotic culture is still present in numbers high enough to be marketed as such. The microencapsulation would seem to offer a good technological alternative for use in the cheese industry [6] receiving considerable interest. However, this increases the cost [7]. Based on the level of probiotic bacteria needed to provide a health benefit it should be possible to lower the amount that needs to be ingested in cheese by up to a factor of 10^3^ compared with other fermented dairy foods or when consumed as supplements [8]. The panoply of probiotic cheese available will increase significantly in the near future, although many of these will not be sold on the market even if they do have additional advantages such as lower fat or cholesterol content because they will not have a great flavour nor will they show desirable characteristics, for example, in rheology.

Most studies of strains being used as probiotics are based on functional properties and less knowledge is available concerning their capacity to withstand stress related to food manufacturing and storage. Cheese as a probiotic food carrier represents a good choice for the dairy industry showing potential advantages over other dairy fermented products but it is also a technological challenge at the same time. Strain viability and maintenance of desirable characteristics during processing steps and storage are a must to assure a beneficial effect [9]. In this review some of the aspects related with the probiotic strains and food technology parameters involved in the elaboration of probiotic cheese are discussed. Some trends and perspectives for the near future are also discussed.

## 2. Probiotic Strains: Culture Production and Viability in Cheese

In practice, probiotic* Lactobacillus* spp. and* Bifidobacterium* spp. are the most common microorganisms included in cheese. Because of their physiology, they are very well suited to this matrix. Of course bacteria other than these may play essential roles in probiotic cheeses [10]; therefore other microbial candidates are expected to and will surely increase the number in the future (see Section 4).  Table 1 shows a noninclusive list of the most relevant species/subspecies used or to be used as probiotics in cheese.

It is important to emphasize that an initial and frequently serious problem for any probiotic strain to be included in a dairy food elaboration is the large-scale biomass production [11]. Although it is not an aim of this review to focus on the probiotic production, it is noteworthy to mention that scale-up has to solve appropriate fermentation processes, adequate inexpensive ingredients, and growth conditions as well as tests for the presence of possible allergens. The biomass is then concentrated by centrifugation or ultrafiltration and preserved using freeze-drying steps and cryoconservants to maximize later cell recovery. Final steps include proper milling and packaging.

Despite the fact that a particular strain of interest for cheese making can be available in sufficient amounts to cover industrial requirements, it has to be stable irrespective of whether it is designed as a starter culture or a probiotic supplement or an adjunct culture. In fact, they all have many properties in common since they obviously can play different roles. Starter cultures are used at one or more stages in the cheese manufacturing process, which develop the desired metabolic activity during the fermentation or ripening which confer unique characteristics mainly taste, aroma, colour, texture, safety, preservation, nutritional value, and, perhaps, possible health benefits according to the definition of microbial food culture by the European Food and Feed Cultures Association (http://www.effca.org/).

One of the traditional trends in cheese research has been to screen among the existing starter or nonstarter strains and determine whether they could have potential health benefits. The survival of the autochthonous microbiota of samples collected during Pecorino di Carmasciano cheese manufacturing was evaluated when a model mimicking the GIT was used. One of the conclusions was very suggestive; the bacterial survival appeared to be more affected by experimental conditions than strain inherent; thus while some strains showed an acceptable survival when resuspended in skim milk, but not in ewe's milk, the opposite was seen for others [12]. The results support the idea that a screening among autochthonous bacteria with this aim is feasible and possibly useful. However, probiotic strains are most frequently of intestinal origin where they are expected to exert health benefits.

At first glance, the use of GIT strains might be considered a handicap for cheese making since it is hard to manufacture specific cheeses with peculiar and genuine properties when large amounts of adjunct cultures are added. GIT strains are commonly oxygen sensitive and complex nutrients demanding. This represents one of the important reasons why for a new strain it is essential to show reasonable survival behaviour when added during the cheese manufacturing process and also to check effects on carbohydrate, protein, and fat usage as well. A good survival of probiotic microorganisms in simulated gastrointestinal conditions of probiotic strains added to cottage cheese was found as well as a good metabolic behaviour and the generation of potentially antioxidant peptides and antilisterial activity [13].

There are techniques adapted to enhance the viability of probiotic bacteria in cheese including the selection of oxygen-tolerant, acid-tolerant, and bile-resistant strains, but it must always be kept in mind that one of the most important aspects, from the food technology point of view, is the need to develop good sensory properties without changing textures or flavours. It is clear that many of the properties are inherent to the particular strain, but long-term industrial processing and storage conditions may influence them. Thus both technologically relevant and functional properties should be taken into consideration in quality-control measures [14].

Cheese compared to other fermented products shows a better buffering capacity for probiotics, less water activity (usually >0.90) depending of the ripening time, and a low storage temperature (4–8°C) with a storage time of weeks or even years. These values are very variable according to the type of cheese considered; that is, water activity (*a*_*w*_) during first stages of cheese manufacture is >0.99, which it is suitable for the growth and activity of the starter culture. After whey drainage, salting and during ripening the prevailing *a*_*w*_ levels are increasingly lower and below the optimal requirements for most starter bacteria. Therefore, more than likely *a*_*w*_ levels contribute to the control of their metabolic activity and multiplication. Some lactic acid bacteria (LAB) generally display higher *a*_*w*_ than other cheese bacteria such as* L. lactis*,* S. thermophilus*,* L. helveticus,* and* P. freudenreichii* ssp.* shermanii* (displaying optimal *a*_*w*_ values of between 0.93 and 0.98) [15]. High values should promote cell counts probiotic maintenance at least for fresh or short ripened cheeses. The water loss through evaporation, the salting stage, and the hydrolysis of proteins and triglycerides cause a fall in *a*_*w*_ throughout ripening. The solution to control moisture loss is usually the increase of the relative humidity in the ripening chambers or the packaging in wax or plastic. Moreover, the lack of homogeneity could be a problem as different in-depth areas of the cheese show different *a*_*w*_ values. It is well known for many cheeses such as Camembert that the pH increases continuously during ripening from acidic to slightly alkaline, whereas *a*_*w*_ decreases displaying a wide range of values depending on the particular nonstandardized conditions used. In general, brine-salted hard and semihard cheeses show higher values towards the centre, whereas in cheddar cheese no loss of moisture and no change in *a*_*w*_ values occur since the salt is uniformly distributed in the cheese and it is vacuum packed. Most probiotic cheeses are protected by a proper wrapping to ensure moisture content; this circumstance does not in any way exclude the requirement to systematically analyse these parameters for the many different kinds of the proclaimed probiotic cheeses present worldwide.

## 3. Cheese Making and Probiotics

Cheese manufacture is essentially a dehydration of milk combined with other preservative effects, such as culturing, salting, packaging, ripening, and/or storage. Some major hurdles associated with the addition and viability maintenance of probiotic bacteria in the development and processing of functional cheese are discussed here.

### 3.1. Milk Culturing, Probiotic Inoculation, and Cheese Processing

Milk is where the history of cheese begins. In large-scale cheese manufacture, the milk is generally pasteurized, for example, 73°C for 15 seconds. It has been described that nonstarter lactic acid bacteria (NSLAB) can survive pasteurization at low numbers and slowly grow during cheese ripening up to 10^6^-10^7^ CFU/g, depending on the ripening period and temperature [16]. Most NSLAB species are lactobacilli, pediococci, and micrococci. This is interesting because at least in the first two cases diverse species involved include NSLAB as well as probiotic strains, that is,* L. casei*,* L. paracasei*,* L. plantarum*, and so forth. The use of both kinds of strains could improve flavour intensity of the cheese and provide suitable technological properties such as longer storage periods. The addition of* L*.* plantarum* I91 and* L*.* paracasei* I90 as selected strains of NSLAB exerted a technological and probiotic role in the elaboration of cheese showing satisfactory properties for their use as adjunct cultures, achieving the dual role of being secondary starters and probiotic cultures [16].

An alternative to the traditional thermal milk treatment is a hyperbaric treatment through high-pressure homogenization (HPH). This approach was used in the elaboration of Crescenza cheese using* S*.* thermophiles* as a starter and commercial probiotic lactobacilli [17]. The authors carried out compositional, microbiological, physicochemical, and organoleptic analysis from 1 to 12 days of refrigerated storage (4°C). No significant differences were found in comparative analysis with adequate cheese controls for gross composition and pH. On one hand, there was a good technological behaviour since HPH-milk increased the cheese yield to about 1% and positively affected the viability during the refrigerated storage of the probiotic bacteria. On the other hand a significant positive effect on free fatty acids release and cheese proteolysis was observed. No significant differences were found for diverse sensory descriptors.

The use of ultrafiltered milk (UF) in cheese making is reasonably well established and attracting considerable worldwide attention. Probiotic Iranian ultrafiltered feta cheese was produced by inoculating the heat treated retentate with a probiotic* L. casei* strain [18]. UF cheese has been traditionally produced as full-fat cheese, but lately UF cheeses are low-fat dietetic products. A reduced fat UF cheese was manufactured following the established production procedure by mixing milk protein powder, skim milk, and cream with adjunct probiotics [19]. The authors found enhanced secondary proteolysis, maintenance of adjunct culture population over a two-month ripening period, a remarkably improved aroma compared with the control, and an overall high count of probiotic* L. acidophilus* throughout the ripening period.

A combined addition of probiotic bacteria and starter culture requires testing appropriate proportions to solve viable probiotic loss during draining. Probiotics can be added as a primary starter or adjunct culture. In the first case, the low capability of probiotics to generate lactic acid during fermentation could be considered a handicap while a joint addition would be a more suitable solution.

Two-step fermentation for cultured dairy products has been shown to be effective in increasing the viability of probiotic bacteria by allowing probiotics to become dominant prior to the addition of the starter cultures. Since starter bacteria could produce inhibitory substances against probiotic bacteria and grow quicker during fermentation, the viability of probiotics could be reduced. Fermentation with probiotic bacteria initially for 2 h followed by fermentation with starter cultures may be helpful in improving the viability of the former and result in higher counts. This has allowed the probiotic bacteria to be in their final stage of lag growth phase or early stage of log phase and thus could dominate the microbiota, resulting in higher counts. The initial counts of probiotic bacteria have been found to increase by four to five times in the product elaborated by the two-step fermentation process. The probiotic bacteria could also be totally added at the end of fermentation [14].

Two types of inoculation methods were compared [20]; in one type of experimental cheese, probiotic bacteria were directly added to the milk as a lyophilized culture, while in the other they were preincubated in a substrate composed of milk and milk fat and then added to the milk. As a result, the direct addition as a lyophilized culture was considered more efficient as direct addition was easier, quicker, and less vulnerable to contamination. Although preincubation in the substrate increased the probiotic population in the inoculum by almost one log cycle, which can be considered more cost-effective for industry, the addition of probiotics after preincubation in the substrate did not improve their survival during cheese ripening. The substrate did not only enhance the protection of probiotic bacteria; but it was also a more complex methodology than direct addition of lyophilized culture. Firstly, it was more time consuming and secondly preincubation could be a sensitive step when considering issues related to contamination and phage attack.

In the case where the probiotic is added later than the starter, a cooling step is normally included to reduce both metabolic activities; later coagulant agents such as lemon juice, plant rennet, or proteolytic enzymes such as chymosin (rennin) or even mold derived are added. The coagulation then occurs under controlled temperature conditions when the previously mentioned enzymes display optimum activity. The slightly acidic environment under which LAB releases enough lactic pH is reduced creating an appropriate environment for optimum activity of rennin. As the processing continues, lower values create a nonsuitable atmosphere for unwanted microorganisms.

There are some serious problems to solve in order to obtain survival improvement of probiotics and procedures to help probiotics overcome the above-mentioned hurdles. Probiotics are also very often placed into cheese in slightly different ways from those present in industrial protocols. One clear trend is the microencapsulation (ME) of probiotic bacteria. Alginate-based or other types of coatings are valid carriers of probiotics and prebiotics because of their nontoxicity, biocompatibility, and low cost [21]. For example, cells immobilized in calcium alginate gels have been added to Crescenza cheese in an effort to improve the survival of bifidobacteria in the final product [22].

Lamb rennet pastes containing encapsulated* L. acidophilus* and a mixture of* B. longum* and* B. lactis* were designed for the manufacture of Pecorino cheese from Gentile di Puglia ewe's milk [23]. On one hand,* L. acidophilus* retained its viability for a few days and then showed a quick reduction. On the other hand,* B. longum* and* B. lactis* showed an initial death slope, followed by a tail effect owing to acquired resistance. After an initial period in which the lowest levels were observed, the highest levels were reached after one month of ripening and then remained so until the end for* L. acidophilus*, whereas bifidobacteria underwent a decrease of about 1 log CFU/g. Greater enzymatic activities and positive correlation were found between enzymatic activities and water-soluble nitrogen and proteose-peptone in probiotic cheeses because of its release from alginate beads.

In another interesting study a fairly good survival rate was obtained using the alginate-microencapsulation of a probiotic* L. paracasei* ssp.* paracasei* strain during the manufacture of Mozzarella cheese, a* pasta filata* cheese in which the curd was heated to 55°C and stretched in 70°C-hot brine followed by a 6-week storage at 4°C [8].

The probiotic cheddar cheese deserves a special mention as it is currently the most widely produced and consumed hard cheese in the world. Different reports indicate, yet again, that different species/strains of* Lactobacillus* and* Bifidobacterium* display different survival ability, although some reports clearly describe that factors such as salt, oxygen, and temperature negatively affect the viability [24].

An improvement of survival from freezing and simulated gastrointestinal conditions of* B. longum* 15708 was confirmed by ME in alginate beads although salting of the curd had a negative effect [25]. The authors observed a 100 times lower viability loss with ME during the technological steps. However, there was a 2 log CFU/mL reduction after 21 days of storage, still unsuitable levels for commercial leading purposes. A clear conclusion is that some probiotics could be highly sensitive cultures. Results were promising since polymers produced showed a relatively good survival as compared to* B. longum* free cells with 3-4 log CFU/mL reductions in addition to an increased resistance to simulated gastric and intestinal environments by a factor of 30.

A sensory acceptance for any food must be ensured after an initial interest for health claims by the consumer [26]. Thus, it is important to use probiotic bacteria with mild acidifying ability to prevent an excessive formation of organic acid. An excessive proteolysis is also linked to inadequate storage and ripening temperature, which in turn could change the organoleptic properties of the final product [4].

Probiotic cultures may change the flavour or texture sometimes in a positive way, as has been reported in petit-Suisse cheese [27]. It has been published elsewhere [28] that probiotic bacteria should remain viable but not metabolically active as reported with* B. longum* in cheddar cheese without affecting their sensory properties. It is of course possible to develop probiotic dairy foods with similar acceptance as conventional products. The addition of increasing amounts of probiotics is apparently a simple solution to ensure a proper microbial viability. Either way sensorial analysis has to be made along with other analyses as well. No negative effect on gastrointestinal welfare was observed in an animal model after an intake of probiotic semihard Edam-type cheese containing* L. plantarum* at a daily dose of 10 log CFU for 3 weeks but the consumption of 100 g/d caused hard stools from the second week of the assay [29].

The processing of cheese can also be affected when a high level of supplementation is used. Some reports have shown a few negative sensory effects with a probiotic* L. acidophilus* strain during the processing of Minas fresh probiotic cheese when high counts (>9 log CFU/g) were present throughout shelf-life [30]. The probiotic cheese presented lower pH values and a greater production of organic acids but lower scores for appearance, aroma, and texture. The same authors have reported that the development of a probiotic cheese requires the handling of different technological options to guarantee a proper functionality throughout during shelf-life.

Some manufacturing procedures include a heating or a cooking stage of the curd. A heating generally between 37 and 45°C affects the rate at which whey is expelled as well as the growth of the starter culture. Curds and whey are often stirred to separate particles. Once curd particles have become firm and a correct acid development has taken place, the whey is removed allowing the curd particles to join together. Once the curd has reached the desired texture it is broken up into small pieces to enable it to be salted in cheeses such as cheddar. Milling the curd can be carried out either by hand or mechanically. In Fior di Latte cheese manufacturing after a proper curd-ripening phase, the drained curd is stretched in hot water. A previous selection of heat-resistant probiotic lactobacilli resulted in a good choice to obtain adequate survival rates under heat conditions, which mimicked the stretching of the curd. After a screening to heating resistance (65 or 55°C for 10 min) in 18 probiotic strains the addition of specific probiotic heat-adapted* L. delbrueckii* ssp.* bulgaricus* and* L. paracasei* strains enhanced shelf-life and cheese flavour formation [31].

Cottage cheese is an unripened, particulate, and acidic cheese made from skim milk. The curd is cut and heated to 55°C; then a cream and salt dressing is added as well as the probiotic. This procedure appeared to be desirable once the adverse effects of the high scalding step were avoided. It is also important to consider their physiological state in order to form an idea of their survival throughout ripening and/or storage. In terms of the growth curve, microbial cells between the late exponential and the stationary phase are the favourite option and the preparation of a previous substrate to inoculate the strain may be sometimes beneficial.

The form of the probiotic inoculants and their viability and maintenance represents an important technological challenge. Milk powder containing a probiotic* L. paracasei* strain as adjunct starter was spray-dried during cheddar cheese manufacturing with a low loss of viability and no adverse effects after three months of ripening [32]. In a semihard cheese the use of either a freeze-dried powder dispersed in milk or a substrate containing milk and milk fat has been proposed for improving survival of probiotic bacteria [33].

### 3.2. Salting and Packaging

It is well known that probiotic bacteria are sensitive to high salt concentrations. The viability of probiotic bacteria decreased drastically in cheeses with salt concentration of over 4% [18]. This implies the need to optimize production conditions in order to incorporate functional characteristics. Almost without exception a dry salting of the milled curd, a surface dry salting after moulding or a brine immersion [34] are used in cheese elaboration after rennet coagulation and curd formation to enhance taste of the curd, safety, and shelf-life. Possible solutions include microencapsulation, cell incubation under sublethal conditions, and careful strain selection. This has to be carried out without negative effects on texture, aroma, and/or acceptance by the consumers. An excellent review on the encapsulation applications in probiotic dairy products and cheese technology is now available [35].

The viability of encapsulated probiotic* B. bifidum* BB-12 and* L. acidophilus* LA-5 was studied in white brined cheese and using Na-alginate by either an extrusion or an emulsion technique. The authors found effective both techniques being the probiotic population higher than the therapeutic limit [36]. The counts for nonencapsulated and microencapsulated probiotic bacteria decreased approximately by 3 and 1 log, respectively. In other cases, results were not so promising. The microencapsulation of probiotic* L*.* acidophilus* DD910 and* B. lactis* DD920 in calcium-induced alginate-starch capsules did not improve their viability in a Feta cheese matrix during storage in brine solution, possibly because of the high salt concentrations [37].

While the viability of probiotics in dry salted cheese varieties has been well documented [38], limited data are available on the probiotic viability in cheeses salted with NaCl and KCl mixtures. New salting procedures include the possibility to substitute at least partially NaCl with KCl. This was shown in Akawi cheese with probiotic bacteria for 30 days of storage at 4°C without apparent significant differences in sensory attributes among experimental Akawi cheeses at the same storage period [39]. The addition of KCl enhanced syneresis in probiotic Iranian Feta cheeses (3% salt and 3-month ripening period) and only those with a 25% replacement by KCl had similar sensory acceptability to those containing NaCl alone [3]. Very similar results were previously reported in Minas fresh cheese [38].

Most probiotic strains are microaerophilic and anaerobic. For this reason permeability to oxygen exposure during manufacturing and storage is an important issue and to choose a suitable packing and vacuum system is relevant. Probiotic cheese is usually packaged in plastic films with low permeability to oxygen or by using vacuum based procedures. An interesting review on packaging systems has already been published [40]. For the elaboration of Turkish white cheese best scores in flavour and texture were detected when probiotic cheese (including* L. acidophilus*) was vacuum packed following salting compared to the control stored in brine following salting [41].

### 3.3. Ripening and Storage

The ripening process is example of a complex biofilm development in which microbiological and biochemical changes occur in the curd mainly related to the metabolism of residual lactose, lactate, and citrate besides lipolysis and proteolysis [42]. Again a major concern is the probiotic survival over a long essential ripening period devoted to the development of aroma and flavour by the activity of many enzymes. The presence of ripening periods during cheese processing is an additional problem for the stability of a probiotic culture as it is not easily predicted due to the biochemical changes which occur as water activity decreases, often together with further decreases in pH, creating an unsuitable environment for the adjunct cultures. Again a possible solution is the ME and the careful optimization of the ripening conditions.

An additional problem is the proliferation of other nonpathogenic adventitious populations which often become the dominant microbiota in cheese; thus NSLAB establishes a competition for nutrients which makes the quantitative determination of probiotic viability more difficult. Lactobacilli and pediococci represent some of the few contaminant bacteria capable of growing in cheese after manufacture as NSLAB. Both as starter or as NSLAB, these may play different roles in the primary metabolic events during cheese ripening with the proteolysis being the major and most complex biochemical event taking place in most cheese varieties. The casein is broken down into low molecular weight peptides and amino acids. This happens while the cheeses are stored under controlled temperature and humidity conditions.

Generally speaking, probiotic bacteria enzymes act mainly in the secondary proteolysis increasing the aminoacid pool, which contributes decisively to cheese flavour and could be precursors for the synthesis of other flavours or volatile aroma, resulting in off-flavours [43]. On the contrary, lipolytic enzymes have a lower activity when compared to starters and NSLAB. Nevertheless, there are already many examples of probiotic cheeses developed with minimum, even undetectable changes in proteolytic and lipolytic profiles, exerting a positive effect on the overall quality of the cheese as well as the production of bioactive peptides [44].

Cheese companies should provide the required viable probiotic population when the product is sold but also guarantee that this situation continues throughout a time-labelled storage. Probiotic cultures could produce antimicrobial substances thus contributing to inhibit development of pernicious microbiota and subsequently acquire a prolonged shelf-life. In other cases preservative agents such as NaCl are added. Nonadequate temperature storage, for example, 12°C, often present in many retailed food markets could reduce this population and increase consumer rejection due to changes in sensory qualities. Some publications, for example, report survival of probiotics through the relatively hard technological phases of pasta filata cheese production in the elaboration and ripening of Scamorza ewe milk cheese. However, texture and appearance attributes differentiated probiotic from control cheeses [45, 46]. Interestingly enough, the authors described specific criteria that should be implemented in order to monitor the quality of probiotic cheeses.

Studies of cheese as a source of new interesting isolates are quickly accumulating. For example, technologically relevant properties of candidate probiotic* L. plantarum* strains make them especially suitable for dairy products such as the long term survival at refrigerated temperatures, the growth viability in the presence of widely used preservatives, and the acidifying, coagulating, and enzymatic activities [47]. In another study a culture containing probiotic* L. fermentum *strains derived from human faeces was suitable and did not adversely affect Turkish Beyaz cheese quality in the four months of ripening [48].

Suitable probiotic properties can be screened* in vitro* before application; that is, potential probiotic strains from Feta, Kasseri, and Graviera cheeses were tested searching for those showing good levels of *β*-galactosidase, low proteolytic and coagulation activities, and antibacterial activities which could be properly exported to to get improvement during longer storage periods [49].

Other activities present in potential probiotics can be used to obtain prolonged ripening and improved storage such as antifungal and anti-Listeria activities as potential preservatives. This approach could provide useful elements for the development of probiotic adjunct cultures producing natural biopreservatives during food fermentations. For example authors detected antimicrobial and antifungal activities of* L. curvatus *strain isolated from homemade Azerbaijani cheese. These authors evaluated probiotic properties of this strain, as well as its safety regarding antibiotics resistance [50].

## 4. Trends and Perspectives

It is clear that the development of new probiotic cheese varieties and derived products will be the leading force in the near future. Some specific aspects and concerns are as follows.

### 4.1. Nutritional Facts

A leading focus in probiotic cheese development is based on the nutritional facts. There is an increasing demand for* diet* or* light* foods. A good example of this is the study of the influence of sweeteners in probiotic petit-Suisse cheese in concentrations equivalent to that of sucrose. Of great interest is the conclusion that none of the assayed sweeteners exerted negative consequences on the viability either on the starter or on the probiotics [51].

Manufacturing companies are providing consumers with cheese derived products containing reasonable levels of sodium, including natural cheeses, processed cheeses, dips, dressings, and spreads. A leading technology is to replace sodium by potassium chloride, both reduce free water and microbial growth and the onset of pathogens. Traditional potassium chloride contributes to undesirable bitterness, but some formulations present in the market seem to overcome this drawback.

Only a few studies have considered the effect of probiotic adjuncts on fatty acids and conjugated linoleic acid (CLA) composition. It has been reported that LAB can produce CLA from linoleic acid, which is a biofunctional lipid [52].

A positive correlation between the CLA and linoleic acid contents of* L. paracasei* and* L. acidophilus* cheeses was observed when Pickle white cheeses with five different probiotic cultures have been studied [33]. The CLA content during the storage period increased because the lipolysis of free linoleic acid by LAB. Another interesting contribution was the elaboration of a probiotic caprine coalho cheese naturally enriched in CLA as a vehicle for* L. acidophilus* and beneficial fatty acids [52].

### 4.2. Safety Aspects

Strain safety will continue to be a concern especially in relation to young and elderly people. A premarket safety assessment of food microbes is based on four mainstays (establishing identity, body of knowledge, possible pathogenicity, and final use) according to the European Food Safety Authority (EFSA) [53]. A representative example is* Enterococcus faecalis *which includes commensal, starter culture, and probiotic strains [54]. However,* E. faecalis* is a highly diverse species that also includes pathogenic strains. A great emphasis on the importance and challenge of precisely characterizing strains from various sources has been made [55]. In fact, these authors have developed a typing scheme and found that a specific genetic cluster included most probiotic and cheese-derived strains. Therefore, strains clustered to this genetic group are more likely to have potential for safe usage as cheese starters and/or probiotics. Available data does not support inclusion of the genus* Enterococcus* within the Qualified Presumption of Safety (QPS) concept of the European Food Safety Authority (EFSA), as safety aspects cannot be determined at the genus or species level but have to be evaluated specifically for each strain [55, 56]. In these cases it seems to be clear that a case-by-case approach should be adopted.

The finding that enterococci are present as a normal microbiota in the human mammary gland during breast breeding [57] opens new interesting perspectives and the possibility to modify QPS status provided genetic fingerprinting techniques unambiguously guarantee the strain identification.

Pediococci and propionibacteria are also frequently used as cheese starters (mainly Swiss cheese or Emmental) and hence consumed in large quantities with apparently no side-effects. Some species/strains have a long history of apparent safe usage in the food chain and consequently some species/strains will be used for cheese manufacture.

### 4.3. Strain Screening

An area of active research is to search for strains showing desirable characteristics such as the ability to produce antimicrobial compounds, the absence of antibiotic resistances, and the capacity to survive the imposed technological hurdles during cheese manufacture. Almost all studies have been focused on* Lactobacillus* sp. and* Bifidobacterium* sp. but of course other bacteria may play essential roles in probiotic cheeses.

There is a recent and growing interest in the probiotic potential propionibacteria (PAB); they are known not only for their ability to produce propionic acid with antimicrobial properties particularly against fungi but also to produce a variety of bacteriocins as well, with a wider antimicrobial spectrum covering other Gram-positive bacteria including LAB, Gram-negative bacteria, and yeasts and filamentous fungi, in some cases [58]. The dairy species are involved in the ripening of the widely consumed Swiss-type cheeses such as Emmental where their concentration reaches 10^9^ bacteria per gram. The dairy propionibacteria show technological properties very suitable for their uses as probiotics in cheese; that is, they display tolerance to technological stresses such as reconstitution in milk, fermentation of a wide range of carbohydrate substrates, microencapsulation, spray-drying, freeze-drying, and storage at low temperatures. Of particular interest is that the *β*-galactosidase activity remained after withstanding the cooking temperature of Swiss-type cheeses and remained stable during storage at low temperatures.

Propionibacteria in cheese have better tolerance to acid challenge than free cultures and they produce propionic acid, a natural biological acid which benefits the bifidus microbiota and displays a good constitutive survival under digestive stress [59]. However, supplementation with dairy propionibacteria has mainly involved mixtures with probiotic bacteria from other genera. There are available dietary supplement capsules designed to maintain the intestinal ecosystem balance which includes two* P. freudenreichii* strains (Sécuril, http://www.swansonvitamins.com) on the market. The use of propionibacteria as adjunct probiotic or in combination with LAB and/or bifidobacteria is a matter of time. It has been reported that a cheese containing a mixture of probiotics (lactobacilli and* P. freudenreichii* ssp.* shermanii* JS) reduces the risk of high yeast counts, especially* Candida* sp. in the elderly [60]. An excellent recent review on probiotic propionibacteria is available [61].

With regard to* Pediococcus*, only* P. acidilactici* and* P. pentosaceus* are relevant dairy strains found in cheeses as adventitious cultures occasionally used during cheese manufacturing. However, there is a potential interest in some of their properties such as the ability to produce antimicrobial compounds (pediocins) and the modification of the texture due to their capability of producing exopolysaccharides. Some probiotic candidates produce lactate crystal formation through the formation of a mixture of L- and D-lactate isomers which is normally considered as a cheese defect. Furthermore, the addition of pure bacteriocins has so far only a few and limited authorized uses in foods. In 1988, nisin produced by* Lactococcus lactis* received the US-FDA approval as food additive for the first time and is being used in the European Union in some cheeses.

Pediocin PA-1 from* Pediococcus* is now on the market. The use of pediococci producing pediocin PA-1 has the potential to be used to improve sensorial properties and to avoid development of undesirable microbiota. For example, Danisco commercializes in lyophilised* P. acidilactici* (Choozit Lyo. Flav 43) to be included as adjunct starter in the elaboration of cheddar cheese and other semihard cheeses to potentiate aroma and flavour [62]. Other strains will be added in the near future in a similar way.

### 4.4. Daily Intake

A relevant problem for cheese acting as carrier of probiotics results from the high fat and salt content and the relatively low recommended daily intake. The concentration of probiotics in cheese should be about four to five times higher than in other dairy fermented products such as yoghurt. However, this does not apply to fresh cheese, such as Cottage cheese, which can easily be adjusted to low fat and salt contents and for which recommended daily intake is higher. Low-fat fresh cheese may thus serve as a food with a high potential to be applied as a carrier of probiotics. In a recent report flavour profiles of reduced-fat and semihard cheeses manufactured with* L. paracasei* ssp.* paracasei* (strains CHCC 2115, 4256, and 5583) were analyzed [63]. The authors observed that reduction in fat content did not affect the population of* Lactobacillus* strains reaching 10^8^ CFU/g throughout the storage period. Because reduced and low-fat cheeses contain more moisture and are generally produced using lower cooking temperatures, lactic acid bacteria are capable of growing high populations.

### 4.5. Synbiotic Cheese

One of the latest trends is to add simultaneously prebiotic and probiotic to fresh cheese (the so-called synbiotic cheese). Knowledge on synbiotics is fairly limited despite the fact that they will probably be one of the next most featured subjects in probiotics research.

In the Fior di late cheese the elaboration was carried out after a proper curd-ripening phase using an edible coating as carrier of probiotic (*L. rhamnosus*) and fructooligosaccharide (FOS). The combination improved the final taste of the product extending its shelf-life [64]. The addition of FOS and inulin did not affect probiotic viability growth and viability of* L. casei* 01 and* B. lactis* B94 during manufacture or a two-month ripening period. However, they generated an improved free fatty acid profile. Another example is the recent work on tagatose which is an epimer of fructose naturally present in small amounts in dairy products [65]. This low reduced-calorie monosaccharide enhanced the growth and probiotic functions of* L. casei* 01 and* L. rhamnosus* strain GG.

Transcriptomic studies and quantitative real-time polymerase chain reaction tests showed induction of a large number of genes associated with carbohydrate metabolism including the phosphotransferase system (PTS) in* L. rhamnosus *strain GG. This is the first confirmation of the catabolism of tagatose by a lactobacilli strain as a prebiotic substrate via tagatose-specific PTS. This study reflects the kind of molecular studies to be possibly demanded in the near future to highlight the potential application of a synbiotic partner in functional dairy foods such as yoghurt and cheese.

### 4.6. Cheese and Microbiota Behaviour

The study of the survival of autochthonous microbiota in cheese to select those with potential ability to arrive metabolically active to the colon will continue being another active area of research. An example of this has been described earlier [12]. The isolation and screening of microorganisms from cheese have been the most powerful means to obtain useful and genetically stable strains and will be so in years to come. Thus, many efforts are being made to screen NSLAB from cheese elaborated with raw milk searching for high tolerance to the different hostile technological processes. This occurs in the elaboration of Parmigiano Reggiano cheese [66] in which NSLAB fraction comes from untreated milk and it is not a part of the normal whey starter. This fraction is particularly attractive as a bioreservoir for potential probiotic strains suitable to survive to GIT condition.

There are many probiotic cheese varieties available on the market and it is essential to verify the behaviour and the performance of the microbial cultures in this environment. In fact, technological control processes and adaptations of the existing manufacturing ones are usually necessary. Testing parameters are decisive for the marketing of the product, such as organic acid profile, typical aroma compounds, or other sensorial attributes.

### 4.7. Health Benefits

The development after in-depth studies of products designed to improve wellness will be strongly supported. For example, some publications indicate the potential function as a dietary item of the probiotic cheese with specific* L. plantarum* [67] or* L. acidophilus* and* Bifidobacterium longum* [68] strains to reduce the risk of cardiovascular diseases. In the first case, the inclusion of* L. plantarum* K15 in cheddar cheese lowered cholesterol effects in a mouse model. In the second case, a popular Brazilian probiotic fresh cheese (Minas Frescal) attenuated exercise-induced immune suppression in Wistar rats thus opening an alternative to enhance the immune system and to prevent infections.

Recent studies on probiotics are leading to their administration combined with diets focused on the control of the metabolism of carbohydrates and lipids and the amino acid turnover. Of particular interest are the results found by Sharafedtinov et al. [69]. They found that the consumption of a hypocaloric diet supplemented with protein-rich full-fat cheese resulted in the lowering of blood glucose levels by 18% without increasing levels of total cholesterol, low density lipoprotein, or triglycerides. These authors concluded that the combination of a hypocaloric diet supplemented and probiotic cheese could help to reduce body mass index, arterial blood pressure, and the risk of metabolic syndrome in obese patients with hypertension.

## 5. Concluding Remarks

Although a number of ready-to-use probiotic strains are now commercially available worldwide as probiotics in cheese, new strains claimed as probiotic or beneficial adjunct cultures will surely increase the present-day list. Main considerations have to include the ability to grow in different economical media such as milk or cheese whey to be available in large quantities and to be adapted to the technological challenges (mainly high temperature and salt content) involved in manufacturing. Many sensorial and nonsensorial aspects are involved in consumer acceptance and have to be considered: flavour texture and of course, last but not least, the price.

The dairy sector has a major advantage in the probiotic foods sector and cheese offers initial advantages as a probiotic carrier. Thus some steps forward seem to be the development of new varieties, the incorporation of new probiotic and well characterized strains, or the manufacture of synbiotic cheeses.

Although it seems apparently obvious, it is noteworthy to mention that the effects on health improvement are strain dependent. No probiotic strain is available and capable of providing all the benefits previously reported [2]. A probiotic cheese is a food not a medicine, which means it is not an alternative treatment for any health condition. Consult your doctor.

## Figures and Tables

**Table 1:** Most relevant species/subspecies of probiotic bacteria successfully added to cheese. ^*^Species including candidate strains or of possible potential use as probiotics. P = *Propionibacterium*. Data collected and adapted from Karimi et al. [4].

*Lactobacillus *	*Bifidobacterium *	Others (∗)
*L. acidophilus *	*B. animalis *	*Enterococcus faecalis *
*L. casei *	*B. animalis *ssp.* lactis *	*E. faecium *
*L. casei *ssp.* pseudoplantarum *	*B. breve *	*Lactococcus lactis *
*L. casei *ssp.* rhamnosus *	*B. infantis *	*Leuconostoc paramesenteroides *
*L. delbrueckii *ssp.* bulgaricus *	*B. lactis *	*P. freudenreichii *ssp. *shermanii *
*L. delbrueckii *ssp.* lactis *	*B. longum *	*Streptococcus thermophilus *
*L. gasseri *		
*L. paracasei *		
*L. plantarum *		
*L. rhamnosus *		
*L. salivarius *		
